# Supplementary material for: Comprehensive Evaluation of 202 Cotton Varieties (Lines) and Their Physiological Drought Resistance Response During Seedling Stage
Source: Plants (Basel). 2025 Jun 10;14(12):1770. doi: 10.3390/plants14121770 (PMC12196738; doi:10.3390/plants14121770)
Supplement: Supplementary file 1 [file plants-14-01770-s001.zip › plants-3671535-supplementary.pdf]

Supplementary Materials

Table S1. Standardised treatment values, D-values and drought tolerance classification of 202 varieties (lines) for each trait

Varieties /Lines	Standardised Treatment Values									
	X(1)	X(2)	X(3)	X(4)	X(5)	X(6)	X(7)	X(8)	D Value	Classification
J206-5	1.01	0.20	0.79	0.01	1.01	0.25	0.66	0.84	0.63	I
jiumian20	0.70	0.17	0.58	1.01	0.42	0.21	0.77	0.76	0.52	I
TD1	0.30	0.09	0.12	0.48	0.09	1.01	0.36	0.23	0.40	I
zhongmiansuo27	0.53	0.13	0.18	0.42	0.26	0.15	0.43	0.86	0.40	I
xinluzhong87	0.47	0.13	0.35	0.51	0.22	0.15	0.51	0.77	0.39	II
sumian7381	0.95	0.28	0.01	0.30	0.82	0.09	1.01	0.20	0.39	II
TD2	0.50	0.12	0.63	0.43	0.21	0.12	0.62	0.61	0.37	II
donglannaxiangdahua	0.30	0.09	0.29	0.56	0.10	0.01	0.43	1.01	0.37	II
zhongmiansuo35	0.44	0.15	0.28	0.71	0.25	0.12	0.48	0.66	0.37	II
K7	0.49	0.13	0.34	0.34	0.26	0.23	0.55	0.54	0.36	II
xinshiH16	0.65	0.14	0.36	0.27	0.18	0.06	0.81	0.55	0.34	II
mianbo188	0.01	0.11	0.18	0.60	0.06	0.12	0.26	0.96	0.34	II
jinke21	0.34	0.16	0.37	0.53	0.27	0.11	0.48	0.53	0.33	II
fuquan9	0.17	0.14	0.24	0.69	0.16	0.16	0.41	0.65	0.32	II
zhongmiansuo45	0.42	0.15	1.01	0.50	0.19	0.08	0.70	0.28	0.31	II
jimian5	0.31	0.17	0.31	0.18	0.24	0.14	0.71	0.53	0.31	II
xinshiH12	0.49	0.15	0.66	0.24	0.21	0.10	0.48	0.39	0.30	II
TD3	0.44	0.09	0.26	0.46	0.15	0.05	0.49	0.54	0.29	III
TD4	0.46	0.13	0.39	0.52	0.19	0.09	0.49	0.40	0.29	III

Table S1. *Cont.*

Varieties /Lines	Standardised Treatment Values									
	X(1)	X(2)	X(3)	X(4)	X(5)	X(6)	X(7)	X(8)	D Value	Classification
xinkenK73	0.36	0.14	0.37	0.53	0.17	0.18	0.37	0.39	0.29	Ⅲ
chuangmian58	0.34	0.16	0.38	0.52	0.24	0.10	0.68	0.34	0.29	Ⅲ
chuan2806	0.33	0.14	0.50	0.53	0.17	0.09	0.53	0.41	0.29	Ⅲ
xinluzhong82	0.28	0.12	0.22	0.27	0.15	0.18	0.40	0.53	0.28	Ⅲ
HN1409	0.47	0.12	0.33	0.65	0.20	0.13	0.44	0.32	0.28	Ⅲ
TD5	0.41	0.12	0.27	0.46	0.17	0.08	0.71	0.40	0.28	Ⅲ
xinluzao1	0.13	0.11	0.10	0.45	0.11	0.71	0.28	0.08	0.28	Ⅲ
TD6	0.40	0.12	0.60	0.52	0.23	0.10	0.52	0.25	0.27	Ⅲ
jijiaomian	0.38	0.14	0.27	0.43	0.20	0.11	0.32	0.44	0.27	Ⅲ
TD7	0.54	0.14	0.33	0.44	0.20	0.06	0.77	0.27	0.27	Ⅲ
zhongmiansuo26	0.20	0.13	0.08	0.49	0.14	0.11	0.36	0.56	0.26	Ⅲ
yuanminanxin13305	0.24	0.12	0.27	0.12	0.14	0.10	0.58	0.50	0.26	Ⅲ
jinken1441	0.33	0.14	0.25	0.16	0.21	0.11	0.49	0.42	0.26	Ⅲ
jizi64	0.09	0.09	0.26	0.80	0.09	0.15	0.35	0.42	0.25	Ⅲ
TD8	0.32	0.11	0.31	0.52	0.17	0.07	0.52	0.35	0.25	Ⅲ
TD9	0.20	0.12	0.31	0.53	0.13	0.11	0.35	0.40	0.24	Ⅲ
meifuchangrong	0.40	0.10	0.37	0.49	0.09	0.10	0.42	0.32	0.24	Ⅲ
zhongda4	0.27	0.12	0.35	0.49	0.21	0.17	0.28	0.25	0.24	Ⅲ
xiyu1	0.37	0.18	0.41	0.43	0.22	0.09	0.48	0.19	0.24	Ⅲ
chuangmian512	0.28	0.13	0.33	0.29	0.18	0.10	0.48	0.33	0.24	Ⅲ
huamian935623	0.43	0.13	0.45	0.37	0.32	0.07	0.50	0.14	0.24	Ⅲ
zhongzhi2B	0.24	0.12	0.28	0.39	0.16	0.09	0.31	0.40	0.24	Ⅲ

Table S1. *Cont.*

Varieties /Lines	Standardised Treatment Values									
	X(1)	X(2)	X(3)	X(4)	X(5)	X(6)	X(7)	X(8)	D Value	Classification
zhongmiansuo17	0.26	0.12	0.18	0.49	0.14	0.12	0.27	0.40	0.23	Ⅲ
haixingnaiyan10	0.42	0.13	0.25	0.67	0.18	0.11	0.46	0.19	0.23	Ⅲ
lv5	0.34	0.13	0.26	0.48	0.18	0.08	0.51	0.28	0.23	Ⅲ
xinluzhong79	0.21	1.01	0.22	0.28	0.12	0.06	0.35	0.19	0.23	Ⅲ
zhongmiansuo143	0.17	0.11	0.28	0.16	0.21	0.12	0.27	0.42	0.23	Ⅲ
ashen36	0.26	0.14	0.32	0.47	0.20	0.11	0.41	0.25	0.23	Ⅲ
jinyu6	0.36	0.23	0.41	0.37	0.21	0.09	0.50	0.15	0.23	Ⅲ
TD10	0.35	0.09	0.13	0.44	0.14	0.06	0.44	0.39	0.23	Ⅲ
A41772BBt	0.27	0.11	0.30	0.50	0.22	0.12	0.36	0.23	0.22	Ⅲ
chuangmian58	0.20	0.15	0.30	0.41	0.17	0.15	0.32	0.28	0.22	Ⅲ
mian9001	0.22	0.13	0.12	0.45	0.15	0.11	0.38	0.35	0.22	Ⅲ
yanmin38	0.38	0.12	0.40	0.26	0.16	0.07	0.43	0.27	0.22	Ⅲ
EZ10	0.25	0.15	0.37	0.48	0.15	0.21	0.31	0.15	0.22	Ⅲ
xinhuimian230	0.26	0.09	0.23	0.49	0.16	0.09	0.34	0.32	0.22	Ⅲ
xinluzhong73	0.27	0.12	0.30	0.32	0.16	0.08	0.46	0.30	0.22	Ⅲ
TD11	0.29	0.13	0.38	0.45	0.23	0.10	0.37	0.18	0.22	Ⅲ
xinluzhong52	0.38	0.13	0.33	0.51	0.17	0.05	0.38	0.24	0.22	Ⅲ
TD12	0.31	0.12	0.31	0.67	0.11	0.08	0.36	0.24	0.22	Ⅲ
zhongmiansuo16	0.63	0.16	0.01	0.46	0.29	0.12	0.65	0.01	0.22	Ⅲ
jimian126	0.28	0.13	0.32	0.22	0.21	0.11	0.46	0.22	0.21	Ⅲ
qianhai6	0.50	0.18	0.32	0.46	0.15	0.07	0.40	0.15	0.21	Ⅲ
xinluzhong59	0.36	0.12	0.36	0.32	0.11	0.08	0.31	0.30	0.21	Ⅲ

Table S1. *Cont.*

Varieties /Lines	Standardised Treatment Values									
	X(1)	X(2)	X(3)	X(4)	X(5)	X(6)	X(7)	X(8)	D Value	Classification
shidaK9	0.41	0.20	0.34	0.38	0.27	0.06	0.46	0.10	0.21	Ⅲ
tada1611	0.35	0.11	0.21	0.53	0.19	0.08	0.45	0.20	0.21	Ⅲ
TD13	0.38	0.10	0.31	0.62	0.13	0.06	0.40	0.21	0.21	Ⅲ
mianmian3	0.27	0.08	0.26	0.44	0.16	0.12	0.38	0.23	0.21	Ⅲ
TD14	0.42	0.14	0.33	0.42	0.21	0.06	0.42	0.16	0.21	Ⅲ
kangchongmian5	0.27	0.15	0.26	0.42	0.22	0.16	0.38	0.14	0.21	Ⅲ
zhongmiansuo44	0.15	0.13	0.26	0.57	0.11	0.07	0.31	0.34	0.21	Ⅲ
xin6015	0.42	0.13	0.39	0.31	0.16	0.06	0.44	0.18	0.21	Ⅲ
shengmian2	0.25	0.13	0.26	0.26	0.13	0.10	0.34	0.31	0.21	Ⅲ
jimian2016	0.24	0.13	0.22	0.29	0.12	0.04	0.39	0.38	0.21	Ⅲ
TD15	0.37	0.13	0.33	0.47	0.18	0.08	0.39	0.16	0.21	Ⅲ
ji169	0.43	0.12	0.25	0.60	0.20	0.07	0.36	0.12	0.21	Ⅲ
xinluzhong88	0.31	0.13	0.33	0.31	0.20	0.08	0.48	0.17	0.20	Ⅲ
zheda304	0.38	0.10	0.36	0.49	0.13	0.07	0.36	0.19	0.20	Ⅲ
hemian20	0.23	0.01	0.31	0.58	0.13	0.10	0.31	0.27	0.20	Ⅲ
su1056 I-1	0.48	0.13	0.34	0.42	0.21	0.04	0.52	0.10	0.20	Ⅲ
tada1619	0.20	0.12	0.09	0.01	0.11	0.08	0.33	0.46	0.20	Ⅲ
TD16	0.19	0.09	0.28	0.52	0.17	0.13	0.32	0.21	0.20	Ⅲ
zhongmiansuo21371	0.36	0.13	0.41	0.40	0.18	0.09	0.31	0.13	0.20	Ⅲ
limian12	0.48	0.11	0.34	0.38	0.23	0.04	0.50	0.10	0.20	Ⅲ
nan6	0.38	0.12	0.37	0.53	0.14	0.04	0.54	0.13	0.20	Ⅲ
huimin605	0.22	0.13	0.32	0.51	0.14	0.08	0.39	0.21	0.20	Ⅲ

Table S1. *Cont.*

Varieties /Lines	Standardised Treatment Values									
	X(1)	X(2)	X(3)	X(4)	X(5)	X(6)	X(7)	X(8)	D Value	Classification
ruifeng2	0.21	0.11	0.35	0.59	0.09	0.06	0.44	0.23	0.20	Ⅲ
xinzhi5	0.32	0.09	0.36	0.54	0.10	0.06	0.40	0.19	0.20	Ⅲ
zhongmiansuo40	0.37	0.11	0.37	0.54	0.11	0.05	0.44	0.16	0.20	Ⅲ
23NJH02	0.16	0.12	0.33	0.50	0.14	0.08	0.32	0.24	0.19	Ⅳ
xiluzao12	0.16	0.09	0.30	0.71	0.03	0.07	0.37	0.28	0.19	Ⅳ
TD17	0.33	0.13	0.34	0.46	0.19	0.07	0.38	0.12	0.19	Ⅳ
youzhi8	0.25	0.14	0.39	0.44	0.11	0.09	0.32	0.20	0.19	Ⅳ
huamian532	0.11	0.08	0.18	0.82	0.04	0.12	0.20	0.28	0.19	Ⅳ
xiluzao13	0.26	0.12	0.36	0.55	0.12	0.06	0.46	0.16	0.19	Ⅳ
jimian262	0.31	0.14	0.33	0.32	0.20	0.07	0.36	0.15	0.19	Ⅳ
tada2	0.35	0.11	0.31	0.41	0.13	0.07	0.33	0.19	0.19	Ⅳ
wanmian37	0.17	0.12	0.14	0.42	0.12	0.05	0.31	0.34	0.19	Ⅳ
jiumianK58	0.22	0.13	0.44	0.43	0.18	0.10	0.29	0.13	0.19	Ⅳ
qianhaiC	0.37	0.12	0.41	0.49	0.14	0.05	0.40	0.11	0.19	Ⅳ
zhongmiansuo49	0.30	0.10	0.28	0.47	0.14	0.07	0.36	0.19	0.19	Ⅳ
zhongmiansuo36	0.28	0.14	0.19	0.48	0.18	0.08	0.34	0.17	0.19	Ⅳ
E21525-1	0.12	0.09	0.14	0.84	0.01	0.06	0.22	0.35	0.19	Ⅳ
xiluzao65	0.20	0.13	0.33	0.34	0.10	0.07	0.51	0.22	0.19	Ⅳ
yinmian2	0.15	0.15	0.25	0.47	0.09	0.09	0.21	0.29	0.19	Ⅳ
TD18	0.26	0.13	0.35	0.51	0.13	0.04	0.44	0.16	0.19	Ⅳ
emian39	0.21	0.15	0.28	0.42	0.17	0.10	0.29	0.16	0.18	Ⅳ
Tazan-1	0.37	0.11	0.26	0.54	0.21	0.05	0.32	0.11	0.18	Ⅳ

Table S1. *Cont.*

Varieties /Lines	Standardised Treatment Values									
	X(1)	X(2)	X(3)	X(4)	X(5)	X(6)	X(7)	X(8)	D Value	Classification
hemian18	0.24	0.11	0.33	0.48	0.13	0.07	0.32	0.18	0.18	Ⅳ
changde184	0.23	0.13	0.29	0.47	0.15	0.07	0.40	0.15	0.18	Ⅳ
TD19	0.10	0.12	0.37	0.61	0.11	0.05	0.36	0.22	0.18	Ⅳ
xinhai23	0.13	0.12	0.21	0.52	0.13	0.08	0.32	0.25	0.18	Ⅳ
TD20	0.30	0.11	0.41	0.47	0.12	0.06	0.29	0.15	0.18	Ⅳ
jinken1565	0.16	0.13	0.27	0.34	0.13	0.09	0.29	0.25	0.18	Ⅳ
jiangnanlu1	0.17	0.12	0.38	0.41	0.21	0.08	0.31	0.13	0.18	Ⅳ
tianza26	0.37	0.11	0.35	0.43	0.13	0.06	0.37	0.12	0.18	Ⅳ
caike586	0.24	0.11	0.25	0.58	0.13	0.09	0.24	0.16	0.18	Ⅳ
tianyu1904	0.40	0.14	0.33	0.35	0.24	0.05	0.44	0.03	0.18	Ⅳ
jiyou851	0.22	0.12	0.24	0.52	0.14	0.05	0.45	0.16	0.18	Ⅳ
shandong105	0.27	0.11	0.37	0.50	0.12	0.07	0.37	0.13	0.18	Ⅳ
xinluzao21	0.34	0.13	0.20	0.54	0.14	0.10	0.37	0.08	0.18	Ⅳ
TD21	0.24	0.12	0.26	0.56	0.13	0.08	0.23	0.17	0.18	Ⅳ
TD22	0.13	0.11	0.35	0.20	0.07	0.07	0.26	0.31	0.18	Ⅳ
xinyu7	0.22	0.10	0.32	0.47	0.07	0.10	0.36	0.16	0.18	Ⅳ
jinghuamian174	0.08	0.14	0.17	0.59	0.11	0.05	0.53	0.22	0.18	Ⅳ
jiumianK1829	0.27	0.16	0.32	0.55	0.17	0.05	0.38	0.08	0.18	Ⅳ
X19075	0.21	0.12	0.30	0.19	0.11	0.07	0.34	0.24	0.18	Ⅳ
E21525-2	0.15	0.08	0.20	0.50	0.06	0.12	0.20	0.26	0.17	Ⅳ
ZS061	0.18	0.10	0.25	0.59	0.10	0.10	0.28	0.16	0.17	Ⅳ
su702	0.21	0.13	0.24	0.61	0.08	0.08	0.31	0.16	0.17	Ⅳ

Table S1. *Cont.*

Varieties /Lines	Standardised Treatment Values									
	X(1)	X(2)	X(3)	X(4)	X(5)	X(6)	X(7)	X(8)	D Value	Classification
R8166	0.18	0.12	0.21	0.68	0.10	0.09	0.24	0.16	0.17	Ⅳ
Ari971	0.17	0.13	0.24	0.46	0.14	0.09	0.27	0.17	0.17	Ⅳ
ning523	0.17	0.10	0.24	0.44	0.16	0.09	0.33	0.15	0.17	Ⅳ
jifeng197	0.29	0.56	0.17	0.56	0.06	0.04	0.23	0.08	0.17	Ⅳ
xingjinghua206	0.11	0.11	0.30	0.64	0.10	0.08	0.28	0.17	0.17	Ⅳ
M-8124-1159	0.22	0.10	0.29	0.67	0.12	0.13	0.43	0.01	0.17	Ⅳ
TD23	0.27	0.10	0.37	0.58	0.08	0.03	0.42	0.11	0.17	Ⅳ
Belshinuo	0.23	0.13	0.18	0.54	0.09	0.09	0.32	0.14	0.16	Ⅳ
xinzamian1	0.11	0.13	0.24	0.55	0.14	0.07	0.32	0.15	0.16	Ⅳ
lu22	0.20	0.12	0.25	0.61	0.09	0.06	0.32	0.14	0.16	Ⅳ
xinluzao66	0.07	0.11	0.30	0.47	0.09	0.07	0.27	0.21	0.16	Ⅳ
TD24	0.26	0.09	0.29	0.54	0.13	0.05	0.27	0.12	0.16	Ⅳ
zhongmiansuo30	0.39	0.12	0.41	0.42	0.14	0.03	0.36	0.04	0.16	Ⅳ
ao7	0.40	0.09	0.29	0.54	0.05	0.04	0.29	0.11	0.16	Ⅳ
huayu708	0.16	0.11	0.23	0.57	0.05	0.07	0.25	0.21	0.16	Ⅳ
TD25	0.23	0.14	0.33	0.45	0.15	0.03	0.41	0.10	0.16	Ⅳ
TD26	0.18	0.11	0.31	0.43	0.13	0.05	0.33	0.14	0.16	Ⅳ
TD27	0.21	0.12	0.28	0.46	0.15	0.05	0.38	0.10	0.16	Ⅳ
chuanD45	0.25	0.09	0.36	0.56	0.06	0.05	0.31	0.12	0.16	Ⅳ
taiyuan0237	0.25	0.11	0.20	0.67	0.09	0.06	0.24	0.13	0.16	Ⅳ
TD28	0.21	0.11	0.26	0.61	0.05	0.04	0.34	0.16	0.16	Ⅳ
huamian1543	0.09	0.10	0.21	0.63	0.12	0.08	0.28	0.15	0.16	Ⅳ

Table S1. *Cont.*

Varieties /Lines	Standardised Treatment Values									
	X(1)	X(2)	X(3)	X(4)	X(5)	X(6)	X(7)	X(8)	D Value	Classification
su8908	0.23	0.10	0.34	0.55	0.11	0.05	0.30	0.10	0.16	Ⅳ
caimianzong3	0.22	0.13	0.17	0.44	0.16	0.07	0.34	0.10	0.16	Ⅳ
TD29	0.30	0.11	0.13	0.41	0.14	0.08	0.40	0.07	0.15	Ⅳ
jinggang249	0.15	0.13	0.24	0.53	0.10	0.08	0.22	0.15	0.15	Ⅳ
chuanjian3	0.17	0.11	0.13	0.78	0.06	0.04	0.23	0.18	0.15	Ⅳ
xingjinghua231	0.17	0.13	0.30	0.54	0.12	0.05	0.31	0.10	0.15	Ⅳ
zhongmiansuo19	0.37	0.10	0.31	0.32	0.04	0.04	0.30	0.15	0.15	Ⅳ
haoda2861	0.22	0.11	0.18	0.05	0.13	0.06	0.31	0.22	0.15	Ⅳ
jinfenghe8	0.33	0.14	0.28	0.40	0.12	0.04	0.33	0.07	0.15	Ⅳ
TD30	0.10	0.11	0.36	0.54	0.08	0.06	0.25	0.14	0.15	Ⅳ
HF52UP	0.28	0.09	0.16	0.60	0.10	0.06	0.22	0.12	0.15	Ⅳ
lumian29	0.26	0.10	0.38	0.52	0.07	0.04	0.01	0.16	0.15	Ⅳ
xinluzhong14	0.36	0.11	0.28	0.48	0.08	0.02	0.42	0.05	0.15	Ⅳ
xinluzhong38	0.22	0.11	0.29	0.37	0.08	0.03	0.28	0.16	0.15	Ⅳ
taiyuan02-41	0.24	0.11	0.24	0.40	0.12	0.07	0.22	0.11	0.15	Ⅳ
TD31	0.19	0.09	0.23	0.39	0.16	0.08	0.25	0.10	0.15	Ⅳ
xibeiquyu3	0.13	0.11	0.20	0.42	0.10	0.05	0.27	0.17	0.14	Ⅳ
chuangmian509	0.12	0.11	0.27	0.13	0.07	0.12	0.27	0.17	0.14	Ⅳ
xinluzhong56	0.16	0.11	0.30	0.23	0.08	0.06	0.24	0.17	0.14	Ⅳ
xinluzao22	0.28	0.12	0.20	0.65	0.09	0.03	0.36	0.05	0.14	Ⅳ
TD32	0.05	0.09	0.08	0.48	0.09	0.06	0.21	0.24	0.14	Ⅳ
23NJH03	0.25	0.10	0.14	0.48	0.11	0.04	0.26	0.12	0.14	Ⅳ

Table S1. *Cont.*

Varieties /Lines	Standardised Treatment Values									
	X(1)	X(2)	X(3)	X(4)	X(5)	X(6)	X(7)	X(8)	D Value	Classification
damianling69	0.22	0.10	0.28	0.68	0.07	0.05	0.20	0.06	0.14	Ⅳ
433Bt	0.14	0.12	0.28	0.54	0.10	0.04	0.23	0.10	0.14	Ⅳ
xinhuimian233	0.18	0.12	0.10	0.50	0.11	0.05	0.39	0.11	0.14	Ⅳ
liaomian19	0.23	0.11	0.17	0.77	0.06	0.04	0.25	0.07	0.14	Ⅳ
qianjinmian	0.09	0.09	0.29	0.66	0.05	0.03	0.30	0.12	0.14	Ⅳ
xinluzhong77	0.22	0.12	0.16	0.01	0.12	0.05	0.37	0.16	0.14	Ⅳ
TD33	0.13	0.12	0.24	0.42	0.15	0.03	0.31	0.08	0.13	Ⅳ
huimin602	0.20	0.10	0.31	0.17	0.09	0.04	0.30	0.12	0.13	Ⅳ
caimianlv4	0.09	0.11	0.12	0.58	0.10	0.06	0.24	0.12	0.13	Ⅳ
xinluzhong22	0.22	0.11	0.14	0.52	0.10	0.07	0.20	0.07	0.13	Ⅳ
xinluzhong55	0.26	0.11	0.33	0.20	0.07	0.04	0.23	0.10	0.13	Ⅳ
jixin6	0.27	0.13	0.26	0.51	0.07	0.03	0.21	0.05	0.13	Ⅳ
lu27	0.25	0.11	0.21	0.58	0.05	0.04	0.25	0.05	0.13	Ⅳ
zaozhi2A	0.27	0.09	0.34	0.54	0.04	0.01	0.27	0.03	0.12	Ⅳ
xinluzao20	0.17	0.11	0.17	0.56	0.07	0.04	0.21	0.07	0.12	Ⅳ
jinke255	0.03	0.13	0.22	0.57	0.14	0.04	0.22	0.06	0.12	Ⅳ
xinluzhong67	0.17	0.10	0.24	0.26	0.07	0.05	0.19	0.12	0.12	Ⅳ
lu21	0.21	0.11	0.16	0.62	0.08	0.03	0.26	0.02	0.12	Ⅳ
junmian1	0.07	0.11	0.11	0.83	0.03	0.04	0.21	0.08	0.12	Ⅳ
shihezi874	0.19	0.10	0.19	0.69	0.05	0.02	0.21	0.04	0.12	Ⅳ
chuangmian548	0.08	0.12	0.28	0.42	0.09	0.04	0.19	0.08	0.12	Ⅳ
TD34	0.16	0.10	0.24	0.56	0.06	0.02	0.23	0.05	0.11	Ⅳ

Table S1. *Cont.*

Varieties /Lines	Standardised Treatment Values									
	X(1)	X(2)	X(3)	X(4)	X(5)	X(6)	X(7)	X(8)	D Value	Classification
xinluzhong26	0.14	0.12	0.13	0.58	0.04	0.04	0.22	0.07	0.11	Ⅳ
qianhaiA	0.22	0.08	0.22	0.75	0.02	0.01	0.23	0.02	0.11	Ⅳ
xinluzao31	0.16	0.10	0.10	0.65	0.05	0.03	0.20	0.05	0.11	Ⅳ
zhaofeng1	0.01	0.09	0.21	0.55	0.07	0.04	0.24	0.08	0.11	Ⅳ
jimian16	0.20	0.11	0.09	0.53	0.05	0.04	0.27	0.04	0.11	Ⅳ
TD35	0.01	0.08	0.17	0.63	0.02	0.04	0.10	0.11	0.10	Ⅳ
xinluzao4	0.08	0.08	0.09	0.42	0.03	0.03	0.11	0.03	0.07	Ⅳ

Note: Ⅰ, highly drought resistant; Ⅱ, moderately drought resistant; Ⅲ, drought sensitive; Ⅳ, highly drought sensitive.

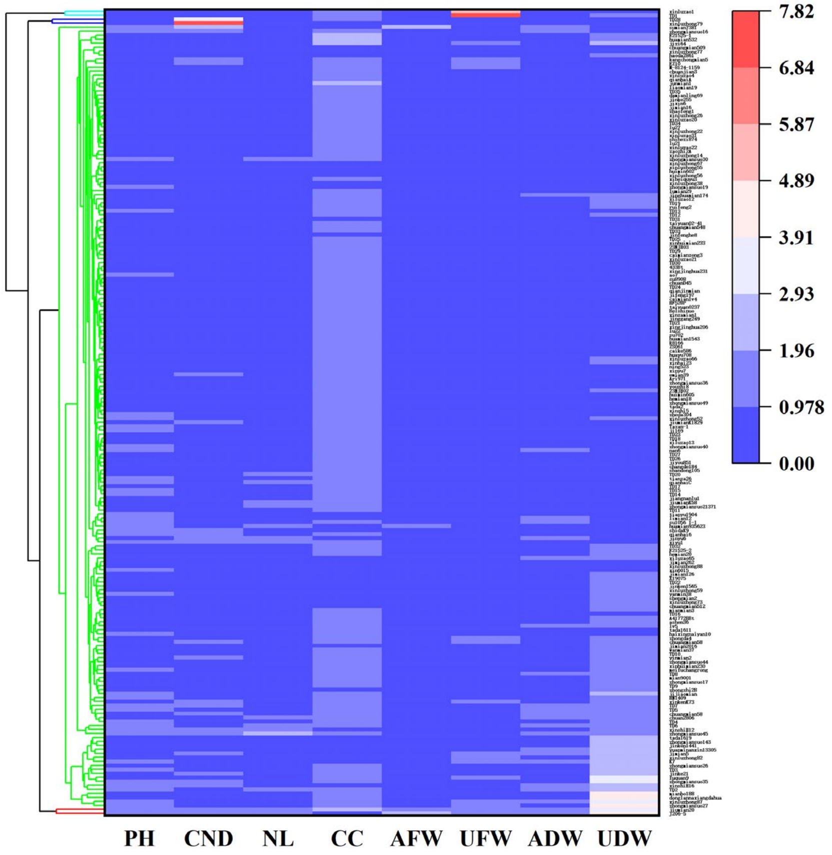

Figure S1: Cluster analysis chart of drought resistance coefficients of 202 cotton varieties (lines). Note: PH, Plant Height; CND, Cotyledon Node Diameter; NL, Number of True Leaves; CC, Chlorophyll Content; AFW, Aboveground Fresh Weight; UFW, Underground Fresh Weight; ADW, Aboveground Dry Weight; UDW, Underground Dry Weight.
